# Medicinal Plants Against Dental Caries: Research and Application of Their Antibacterial Properties

**DOI:** 10.3390/plants14091390

**Published:** 2025-05-05

**Authors:** Marcela Alejandra Gloria-Garza, Gustavo Raúl Reyna-Martínez, Zacarías Jiménez-Salas, Eduardo Campos-Góngora, Miroslava Kačániová, Diana Elena Aguirre-Cavazos, Minerva Bautista-Villarreal, Catalina Leos-Rivas, Joel Horacio Elizondo-Luevano

**Affiliations:** 1 Facultad de Odontología, Universidad Autónoma de Nuevo León, Dr. Eduardo Aguirre Pequeño S/N, Monterrey 64460, NL, Mexico; marcela.gloriagz@uanl.edu.mx; 2 Facultad de Ciencias Químicas, Universidad Autónoma de Nuevo León, Av. Universidad S/N, Cd. Universitaria, San Nicolás de los Garza 66455, NL, Mexico; gustavo.reynamr@uanl.edu.mx; 3 Facultad de Salud Pública y Nutrición, Universidad Autónoma de Nuevo León, Dr. Eduardo Aguirre Pequeño S/N, Monterrey 64460, NL, Mexico; zacarias.jimenezs@uanl.mx (Z.J.-S.); eduardo.camposg@uanl.mx (E.C.-G.); 4 Institute of Horticulture, Faculty of Horticulture and Landscape Engineering, Slovak University of Agriculture, Tr. A. Hlinku 2, 94976 Nitra, Slovakia; miroslava.kacaniova@gmail.com; 5 School of Medical & Health Sciences, University of Economics and Human Sciences in Warsaw, Okopowa 59, 01 043 Warszawa, Poland; 6 Facultad de Ciencias Biológicas, Universidad Autónoma de Nuevo León, Av. Universidad S/N, Cd. Universitaria, San Nicolás de los Garza 66455, NL, Mexico; diana.aguirrecvz@uanl.edu.mx (D.E.A.-C.); minerva.bautistavl@uanl.edu.mx (M.B.-V.); catalina.leosrs@uanl.edu.mx (C.L.-R.); 7 Facultad de Agronomía, Universidad Autónoma de Nuevo León, Francisco I. Madero S/N, Ex Hacienda el Canada, General Escobedo 66050, NL, Mexico; 8 Instituto de Investigación Biomédica de Salamanca, Facultad de Farmacia, Universidad de Salamanca, Campus Miguel de Unamuno S/N, 37007 Salamanca, Spain

**Keywords:** bacteria, caries, ethnopharmacology, extracts, medicinal plants, natural products, plant therapies, preventive dentistry

## Abstract

Dental caries remains one of the most widespread global health concerns, significantly affecting both oral and overall health. Conventional treatments typically rely on chemical-based products which, although effective, are often associated with undesirable side effects such as tooth staining, altered taste, and the development of antimicrobial resistance. As a response, plant-based natural alternatives have gained attention as promising strategies for the prevention and management of dental caries. This review highlights the antibacterial properties of medicinal plants and their potential applications in dentistry, with a particular focus on their activity against a broad range of bacteria and microorganisms involved in oral diseases. Numerous plant extracts and bioactive compounds—including polyphenols, flavonoids, and essential oils—have demonstrated antimicrobial, anti-inflammatory, and antioxidant properties that contribute to maintaining oral health. Although in vitro and in vivo studies support their therapeutic potential, clinical trials assessing long-term efficacy and safety remain scarce. Future research should prioritize the standardization of extraction methods, dosage, and formulations to facilitate the integration of these natural alternatives into conventional dental care practices.

## 1. Introduction

Oral diseases are among the most common diseases around the world that people usually suffer from during their lifetime. The World Health Organization estimates that about half of people suffer from oral diseases [1]. Dental disease is undoubtedly a public health problem and is among the most prevalent diseases globally [2]. The most common disease of the oral cavity, known as dental caries, is a top leader [3] and a major health problem in most industrialized countries, in which many children and adults experience the disease [4]. About 2.4 billion people have permanent teeth decay, and 532 million children are also affected by primary teeth decay. A recent epidemiological study showed that the global amount of caries of permanent teeth cases increased by 46.1% from 1990 to 2019 [5].

The mouth is colonized by 700 to 1000 microbial species, but only some of them are responsible for dental caries [6]. *Streptococcus mutans* is one of the major etiologic agents and a key bacterium that induces caries [7]. *S. mutans* is an acidogenic and aciduric Gram-positive bacterium that naturally inhabits the oral cavity [8]; its cariogenic potential is directly related to its metabolic activity and the development of mechanisms allowing the bacteria to integrate into the dental biofilm and to colonize tooth surfaces [5]. *S. mutans* produce organic acids during diet carbohydrates metabolism, survive in low-pH conditions, and can synthesize extracellular polymeric substances (EPSs). The production of EPSs (glucans and fructans) from sugars promotes bacterial growth, and its adherence to the dental surface, resulting in the formation of a biofilm on tooth surfaces [5]; glucan encourages the accumulation of cariogenic *Streptococci* on the teeth, increasing the pathogenicity of biofilm [9]. Dental biofilm is the primary etiology for dental caries. Unless biofilms are appropriately controlled, they accelerate their physiological heterogeneity and cause a series of complex interactions, tooth demineralization, and systemic inflammation. It is generally accepted that mechanical approaches such as tooth brushing and flossing are fundamental for controlling dental biofilms [10].

To enhance the efficacy of mechanical plaque control and improve daily oral hygiene, antimicrobial agents are commonly incorporated into toothpastes and mouthwashes to inhibit plaque accumulation and biofilm formation in hard-to-reach areas [11]. Oral care products designed to control caries typically contain chemical agents such as fluoride, chlorhexidine (CHX), triclosan, sodium lauryl sulfate, cetylpyridinium chloride, and chlorophyll. Additionally, vitamins, probiotics, and antibiotics—including amoxicillin, erythromycin, and penicillin—are frequently employed in dental practice to suppress microbial growth [12,13,14,15].

While effective, these commercial oral hygiene products have several drawbacks [13]. Most are formulated with synthetic chemicals that may elicit non-biocompatible reactions due to direct contact with the oral tissue [14]. Fluoride, for example, can be toxic if overused; it is not recommended for children under six years old due to risks such as enamel discoloration and structural weakening [13]. Although CHX remains the gold standard among antiseptics, chronic use is associated with adverse effects including yellow-brown staining of teeth, altered taste perception, oral mucosal irritation, desquamative lesions, ulceration, and increased calculus formation, as well as cytotoxicity toward human fibroblasts and osteoblasts [11,15,16]. Triclosan has a limited binding affinity in the oral cavity, potentially reducing its effectiveness. Antibiotic use can also lead to side effects such as nausea, vomiting, and diarrhea [12]. Moreover, the prolonged use of antimicrobial agents [15] contributes to the emergence of resistant cariogenic microorganisms—a challenge that has persisted for nearly a century [16,17,18].

In this context, the discovery of novel antimicrobial agents derived from natural products offers a promising avenue for the prevention and management of oral microbial infections [9]. This review aims to summarize key medicinal plants used against dental caries, emphasizing their antibacterial properties and applications in dental care. Natural products represent advantageous therapeutic alternatives to synthetic drugs, as medicinal herbs possess a wide spectrum of biological activities and are promising sources for the development of new antimicrobial agents [19].

## 2. Search and Inclusion Methods

This panoramic review focuses on the use of medicinal plants in dentistry, with particular emphasis on their potential to prevent and treat dental caries. The review strategy involved compiling scientific articles that addressed medicinal plants and their anticariogenic properties, published in English or Spanish. Relevant literature was retrieved from multiple databases, including PubMed, Google Scholar, SciELO, ResearchGate, and ScienceDirect. The search utilized keywords such as Bacteria, Caries, Ethnopharmacology, Extracts, Medicinal Plants, Natural Products, Plant Therapies, and Preventive Dentistry, which were applied as filters to search all fields. Articles published between 2004 and 2024 were considered.

Following the search, abstracts were carefully reviewed to identify relevant studies. Eligibility criteria included original articles written in English or Spanish that focused on or employed medicinal plants in the context of dental caries or dental biofilm management. The search strategy included the use of Boolean operators *OR* and *AND*, combining terms related to medicinal plants (e.g., “herbal medicine”, “plant extract”, “medicinal plant”) with terms related to dentistry (e.g., “dental caries”, “antimicrobials”, “cariogenic bacteria”). The data extracted from the selected studies provided a comprehensive overview of the current scientific landscape on this topic.

## 3. History and Evolution of Medicinal Plant Use in Dentistry

### 3.1. A Look at the History of Phytotherapy in Dentistry

Nature has long provided an abundant source of herbs for human health; however, the pursuit of modern development has often led to the underappreciation of these potent natural resources. It has been widely demonstrated that herbal medicines—including medicinal herbs, herbal preparations, and phytotherapeutic compounds—offer significant therapeutic benefits for humans [20]. Phytochemicals, the naturally occurring chemical constituents found in various parts of plants, are responsible for the medicinal value of many plant species [21]. According to the World Health Organization, approximately 80% of the global population relies on phytotherapeutic products such as plant extracts, vitamins, infusions, and other similar natural remedies to address a variety of health conditions. This widespread use is largely attributed to the generally high safety margin and tolerability associated with natural agents. Medicinal plants have been shown to possess a broad and specific spectrum of biological activities, including antimicrobial, antioxidant, anti-inflammatory, antifungal, antiviral, and analgesic effects relevant to oral health [13,19].

Since ancient times, plant materials have been traditionally used in various forms—such as chewing sticks, latex or exudates, tooth powders, or mouth rinses—to maintain oral hygiene, prevent dental diseases, and promote overall dental health [13]. The earliest documented uses of herbal remedies in oral health trace back to ancient Indian and Chinese traditional medicine systems. Historical sources mention that Hippocrates recommended a mixture of alum, salt, and vinegar as a mouth rinse. Similarly, the religious manuscript Talmud, dating back approximately 1800 years, advocated the use of “dough water” and olive oil for oral care. The Greek physician Pedanius Dioscorides proposed a mouthwash prepared from wine, milk, and herbal extracts from olive tree leaves and pomegranate [22]. Additionally, as early as 3500 BC, the Babylonians employed chewing sticks derived from the *Miswak* plant (*Salvadora persica*), obtained from the Arak tree—one of the oldest known oral hygiene tools, which continues to be used across various cultures to this day [23].

The main medical books of traditional Persian medicine, such as Kitāb -al-Ḥāwī fī al-Ṭibb by Rhazes (AD 854–925), Al-Qanun fit-Tibb by Avicenna (AD 980–1037), and Zakhīra-i Khwârazmshâhī by Esama’il Gorgani (AD 1040–1136), have a separate chapter about the diseases of the teeth and oral cavity [24].

### 3.2. Development of Plant-Based Treatments

The recent emergence of phytodentistry—an alternative therapeutic approach that harnesses the medicinal properties of plants and herbs to manage a variety of oral conditions—has provided a potentially safe and cost-effective substitute for synthetic drugs [25]. The use of plants in preventing and treating dental diseases dates back centuries. Plant extracts are believed to be effective because they interact with specific chemical receptors within the human body [26].

Oral health is maintained through a delicate balance between commensal and pathogenic microorganisms residing in the oral cavity, where commensal microbiota typically dominate. Oral diseases arise when this microbial homeostasis is disrupted, leading to the overgrowth and colonization of pathogenic species [27]. The most prevalent oral conditions are closely linked to the loss of microbial balance and the formation of dental biofilms [12]. These biofilms can harbor a diverse array of microbial species, including *Candida albicans*, *Candida glabrata*, *Enterococcus faecalis*, *Lactobacillus acidophilus*, *S. mutans*, *Veillonella dispar*, and *Fusobacterium nucleatum*, among others.

Dental caries is now widely recognized as a dysbiosis involving complex interactions among tooth structures, microbial biofilms, and dietary sugars [28]. Among these microorganisms, *S. mutans* is considered the primary etiological agent in the development of dental caries. Therefore, the inhibition of this bacterium’s activity is a key strategy in caries prevention. Numerous studies have investigated the use of herbal extracts against *S. mutans*, highlighting their antimicrobial potential [22]. An overview of recent in vitro studies evaluating the effects of medicinal plants on dental caries is presented in Table 1.

In 2019, Mandava et al. [36] conducted a study to evaluate the level of anticaries efficacy of various medicinal plants against *S. mutans* glucosyltransferases (GTFs). A total of six natural sources were selected as test samples, including four plant species—*Terminalia chebula*, *Psidium guajava*, *Azadirachta indica*, and *Pongamia pinnata*—and two essential oils: clove (*Syzygium aromaticum*) and peppermint (*M. piperita*). Hydroalcoholic extracts of the plants and the essential oils were assessed for their inhibitory potential against GTFs isolated from *S. mutans*. A polyherbal mouthwash was formulated using all six agents, and its inhibitory effect on GTF activity was compared to that of a commercial 5% *w*/*v* chlorhexidine (CHX) mouthwash. All tested samples demonstrated considerable GTF inhibitory activity. Notably, the polyherbal mouthwash exhibited a 95.936% inhibition of GTF activity, compared to 54% observed with the chlorhexidine control, suggesting its potential as a future formulation for combating dental caries [36].

In the same year, Oluwasina et al. [17] formulated herbal toothpastes using the medicinal plants *S. aromaticum and Dennettia tripetala* and latex from *Jatropha curcas*. Phytochemical analysis revealed that *S. aromaticum* and *D. tripetala* contained phenols, flavonoids, alkaloids, and saponins. Gas chromatography–mass spectrometry (GC-MS) analysis identified eugenol, caryophyllene, and phenol, 2-methoxy-4-(2-propenyl)-, acetate as the major constituents in *S. aromaticum*, while *D. tripetala* showed glutaric acid, eugenol, caryophyllene, and 1,6,10-dodecatrien-3-ol, 3,7,11-trimethyl-(E). The study concluded that the formulated toothpastes exhibited significantly greater antimicrobial activity (*p* < 0.05) than commercial formulations, including potent inhibition of *S. mutans*, due to the presence of these bioactive compounds [17].

Valadas et al. (2019) [31] evaluated the optimal antimicrobial concentration of *Copaifera langsdorffii* (copaiba) oil–resin, formulated as a dental varnish, against *S. mutans* in children. The study concluded that copaiba oil–resin exhibits significant antimicrobial activity against *S. mutans* when applied as a dental varnish. This formulation could serve as an effective preventive strategy for children aged 3 to 5 years. However, additional randomized clinical trials are warranted to confirm its antimicrobial efficacy and anticaries potential. Furthermore, long-term studies are necessary to establish its sustained activity and viability as a caries prevention agent [31].

Elgamily et al., in 2019 [32], investigated the antibacterial efficacy of five plant extracts—*C. zeylanicum*, *Curcuma longa*, *Zingiber officinale*, *S. aromaticum*, and *Nigella sativa*—against the growth of *S. mutans*. Among these, the methanolic extracts of cinnamon and clove demonstrated notable antimicrobial activity. These findings support the potential use of these plant extracts as innovative agents in minimally invasive and adhesive dentistry approaches [32].

Previous studies have evaluated the effects of aqueous extracts from five plants—*Petroselinum crispum*, *Eruca vesicaria* ssp. *sativa*, *Beta vulgaris* L. var. *cicla*, *Rumex cristatus* DC., and *Cotinus coggygria* Scop.—on calcium phosphate precipitation, a process believed to reflect the early stages of dental calculus formation in vitro [29]. The effects on calcium phosphate precipitation varied among the extracts. Notably, the extract from the smoke tree (*Cotinus coggygria*) promoted precipitation, while the others inhibited it. These results suggest that some plant-based aqueous extracts may offer protective benefits against dental calculus formation and could be considered for inclusion in toothpaste or mouthwash formulations [29].

In 2020, Khoramian et al. [37] developed a mouthwash containing an aqueous extract of *Teucrium polium* to evaluate its effect on *S. mutans* colonization in the oral cavity. The results showed that *T. polium* significantly reduced *S. mutans* colonization in human saliva, with the reduction persisting even three weeks after discontinuation of the mouthwash. These findings indicate that periodic use of *T. polium* mouthwash may help reduce the risk of dental caries.

Milutinovici et al. in 2021 [19] conducted a comprehensive review of the most commonly used medicinal plants in dentistry, focusing on their active phytocompounds, chemical structures, and mechanisms of action. Many of the reviewed studies evaluated the in vitro and in vivo antimicrobial effects of plant species such as *A. sativum*, *Aloe vera*, *Camellia sinensis*, *Citrus aurantifolia*, *Cocos nucifera*, *C. longa*, *Glycyrrhiza glabra*, grape seed extract, *Hypericum perforatum*, *M. piperita*, *Carica papaya*, propolis, *Melaleuca alternifolia*, and *Thymus vulgaris* on *S. mutans*. The authors concluded that the therapeutic potential of natural extracts is enhanced by the synergistic interactions among their phytochemical constituents. They further emphasized the importance of evaluating these natural compounds in combination with other phytochemicals and conventional drugs to better understand possible synergistic effects. Overall, medicinal plants and their bioactive compounds represent a promising and valuable alternative to conventional dental therapies [19].

Kumar et al. (2021) [16] analyzed the role of antioxidant secondary metabolites in inhibiting the growth of oral pathogens, reducing dental plaque formation, and alleviating symptoms of oral diseases. According to the evidence presented in their review, antioxidant-rich essential oils possess potential as preventive and therapeutic agents for various oral health conditions. While many potential applications of antioxidant essential oils have been identified and their therapeutic efficacy has been validated through both in vitro and in vivo studies, further research is needed to assess their long-term safety and efficacy before they can be widely adopted in clinical practice [27,36].

In the same year, Flemming et al. (2021) [30] explored the structural characteristics of various polyphenols and their interactions with tooth surfaces and the acquired enamel pellicle. Polyphenols, which are natural bioactive compounds, are known for their antioxidant, anti-inflammatory, and anticancer properties. The study highlighted the effectiveness of polyphenols derived from *Cistus incanus* and *Inula viscosa* in reducing the growth of cariogenic bacteria, supporting their use as agents for oral disease prevention [30].

In 2022, Heliawati et al. [14] reviewed 23 studies on the medicinal properties of red betel leaf (*Piper crocatum*), documenting its potential as a natural antibacterial agent for the treatment of oral and dental conditions. Traditionally used for managing toothache and canker sores, red betel leaf contains various secondary metabolites, including flavonoids, polyphenols, tannins, and essential oils. Flavonoids in particular have been shown to inhibit the growth of *S. mutans*. The red betel leaf decoction has antiseptic properties and can function effectively as a mouthwash to combat halitosis [14].

Carvalho et al. (2022) [33] evaluated the anticariogenic activity of three essential oils (EOs) extracted from Brazilian Piperaceae species: *Peperomia pellucida*, *Piper marginatum*, and *Piper callosum*. Their findings suggested that these EOs hold promise for use in oral healthcare products designed to treat dental caries, highlighting their potential for commercialization in phytomedicine. However, the authors emphasized the need for cytotoxicity assays to confirm the safety of their pharmaceutical applications [33].

In 2023, Pourmoslemi et al. [34] investigated the anticariogenic activity of *Verbascum speciosum* in search of new agents for the prevention and treatment of dental caries. The study demonstrated that *V. speciosum* flower extract exhibited strong anticariogenic effects, which were attributed to its antibacterial activity against mutans streptococci and its ability to inhibit glucan synthesis mediated by glucosyltransferase (GTF) enzymes. This extract shows potential as an alternative or complementary component to current anticaries therapies and could be incorporated into dental care products.

In 2023, Shaalan et al. [26] evaluated the antibacterial efficacy of a Miswak-based herbal toothpaste in comparison to a fluoride toothpaste in high-caries-risk patients. The Miswak toothpaste exhibited comparable antibacterial effects against *S. mutans*. However, the release of ions from the Miswak toothpaste was significantly lower than that of the fluoride-containing counterpart. As a result, while Miswak herbal toothpaste possesses potent antibacterial activity, its remineralization potential may be limited due to lower ion substantivity in saliva—an important factor in enamel remineralization.

Table 2 below summarizes several herbal formulations studied for their effectiveness in caries prevention and treatment.

## 4. Medicinal Plants and Extracts Used in Caries Treatment

Dental caries is one of the most widespread oral diseases globally, posing serious health and economic challenges for populations in many countries. When not treated promptly, this condition can lead to complications such as tooth loss, gingivitis, periodontitis, and even oral cancer. Although dental caries is highly preventable in its early stages, the pathology often progresses to more severe outcomes if left untreated. The primary cause of dental caries is the formation of infectious biofilms that adhere to the oral surfaces. These biofilms result from complex interactions between cariogenic bacteria, their metabolic byproducts, saliva, and dietary components, ultimately adhering to susceptible tooth surfaces. Pathogenic biofilms typically include streptococcal species such as *S. mutans*, *S. salivarius*, and *S. sanguinis*, as well as other microorganisms like *Porphyromonas*, *L. acidophilus*, and the yeast *C. albicans* [39].

Several strategies have been employed in the prevention of dental caries, including the use of medicinal plants. Historically, aqueous, ethanolic, and methanolic extracts obtained from various plant parts—leaves, flowers, fruits, seeds, and roots—have been used to inhibit the formation of dental plaque biofilm [40].

For centuries, medicinal plants have played a crucial role in the prevention and management of dental diseases. Initially, humans used to chew on plant leaves, fruits, or roots; later, they began preparing herbal infusions and mouth rinses. In modern dentistry, plant materials are processed to obtain extracts using oils or solvents, with the aim of isolating bioactive compounds that can be incorporated into commercial oral care products targeting dental caries [15]. One common approach to evaluating the anticariogenic potential of these plants involves measuring their in vitro antimicrobial activity against cariogenic bacteria such as *S. mutans* [36].

This section presents a bibliographic review of the most commonly used medicinal plants in the treatment of dental caries. The main active components of these plants and their potential therapeutic applications in dentistry are also discussed.

Maintaining oral hygiene is vital for overall health, and nature provides a vast array of remedies rich in active compounds with demonstrated antibacterial, antioxidant, anti-inflammatory, and anticariogenic properties. Table 3 provides a representative (though not exhaustive) list of medicinal plants used in the treatment of dental caries. The table highlights their global distribution and indicates that various plant parts—ranging from roots to bark and flowers—can be used effectively against caries.

Table 4 shows a diverse array of botanical sources that offer a rich tapestry of compounds, each with unique mechanisms of action against oral pathogens and conditions.

Phenolic acids demonstrate antimicrobial effects against both Gram-positive and Gram-negative bacteria; these compounds are present in *C. canephora*, *C. sativum* and *V. vinifera*, and they display antibacterial properties by reducing oral bacteria such as *S. mutans* by breaking down bacterial cell walls and inhibiting enzymes involved in forming biofilms, also significantly decreasing the viability of *C. albicans* cells, and show potential against *S. mutans* biofilms, combating dental cavities [53,88]. Moreover, *C. sinensis* (black tea) emerges as a preventive measure against periodontal disease, thanks to its polyphenolic compounds like epicatechin-3-gallate, which possess antioxidant, anti-inflammatory, and antibacterial properties [95].

Tannin compounds exert antibacterial effects by disrupting bacterial colonies, primarily through interference with the bacterial cell wall and inhibition of fatty acid biosynthesis pathways. Their activity is enhanced by hydrolysis. The presence of *o*-diphenol groups in tannins enables them to function as iron chelators, depriving microorganisms of this essential nutrient, and further enhancing their antibacterial properties.

Extracts from *A. indica* and *Q. infectoria*, rich in tannins, have demonstrated significant antimicrobial activity against periodontitis and dental caries-causing agents. They effectively reduce the plaque index and the count of bacteria in saliva. Similarly, extracts also containing tannins from various parts of *P. guajava* and *J. regia* have been recognized for their antimicrobial properties. These extracts exhibit antibiofilm and growth inhibitory effects against oral pathogens such as *S. mutans*, *S. sanguis*, and *P. gingivalis*, thereby effectively combating dental caries [59].

*C. intybus* extracts, containing flavonoids or catechins, counteract the virulence factors associated with oral pathogens by disrupting bacterial quorum sensing and inhibiting biofilm formation [86]. *R. officinalis* essential oil, enriched with terpenoids and flavonoids, showcases antioxidant, antibacterial, and antibiofilm properties, contributing to the prevention of plaque accumulation [91]. *C. sinensis* peel extract showcases antibacterial prowess, attributed to compounds like flavonoids [60].

Extracts from *S. asper* significantly decrease colonies of *S. mutans* and exhibit efficacy against *P. gingivalis* and *A. actinomycetemcomitans* [96]. Mouth rinses and toothpaste formulated with *T. chebula* demonstrate antibacterial activity against *A. actinomycetemcomitans* and dental caries-causing bacteria like *S. mutans*, along with possessing anti-inflammatory and antioxidant attributes [92].

*V. macrocarpon* compounds such as anthocyanins, flavonols, and proanthocyanidins impede the colonization of bacteria including *P. gingivalis* and *F. nucleatum* and inhibit the attachment of *P. gingivalis* to various proteins, thus mitigating the risk of periodontal disorders; *L. sidoides* essential oil showcases potent antibacterial activity against cariogenic bacteria and manages supragingival biofilm effectively [61,93,94].

Additionally, neem, Embelia, *J. regia*, *P. guajava* and *C. sinensis*, renowned for their oral health benefits, are rich sources of polyphenols such as tannins and flavonoids, further highlighting their significance in oral care [67,83]. Moreover, *C. sinensis* emerges as a preventive measure against periodontal disease, thanks to its polyphenolic compounds like epicatechin-3-gallate, which possess antioxidant, anti-inflammatory, and antibacterial properties [85,95]. Similarly, *A. sativum* boasts a rich phytochemical profile, including alliin, which converts to allicin, a potent antimicrobial agent targeting oral pathogens [81].

*A. nilotica* stands out with its stem and bark extracts containing quercetin and saponins, which exhibit antibacterial effects by disrupting bacterial cell membranes and inhibiting glucosyltransferase, thereby reducing bacterial counts and controlling dental plaque [41].

The essential oil of these various plants contains diverse arrays and concentrations of terpenes and terpenoids, monoterpenes such as α-pinene, camphene, and eugenol, and sesquiterpenes like caryophyllene and humulene, among many others. Specific compounds found in plants such as *A. racemosus*, *E. globulus*, *E. caryophyllata*, *O. sanctum*, *S. persica*, *T. vulgaris*, and *M. alternifolia* exhibit noteworthy antibacterial properties against various oral pathogens [53,57,64,90]. For instance, *A. racemosus* shows antibacterial activity against *S. mutans* and *L. acidophilus*, primarily attributed to compounds like borneol and myrtanol [18]. *E. globulus* essential oil demonstrates significant antimicrobial efficacy against bacteria commonly found in the oral cavity, inhibiting both planktonic and biofilm growth of *S. mutans* and *E. faecalis* [55,97]. Similarly, *E. caryophyllata*, known for its antiseptic and analgesic properties, exhibits strong antimicrobial activity against streptococci, particularly *S. mutans*. *O. sanctum* essential oil, containing compounds like eugenol and carvacrol, displays antimicrobial and antifungal properties against oral pathogens [53,57].

*S. persica* compounds possess antibacterial, anti-inflammatory, and antioxidant properties, hindering plaque formation and preventing periodontal disease. *T. vulgaris* essential oil demonstrates antimicrobial activity against various pathogenic bacteria and fungi. *N. sativa* essential oil exhibits antimicrobial properties, particularly against *P. gingivalis* and *A. actinomycetemcomitans* [64].

The leaf essential oil of *O. americanum* demonstrates impressive antimicrobial activity against *S. mutans* and *L. casei*, matching the effectiveness of CHX solution. This potent activity can be attributed to the composition of the essential oil, particularly its lauric acid content, renowned for its antimicrobial properties. Derived from the leaves of *O. americanum*, this essential oil exhibits significant antimicrobial effectiveness against cariogenic bacteria, notably *S. mutans* and *L. casei*, in both planktonic and biofilm cultures; it demonstrates a comparable ability to CHX solution in reducing bacterial counts [66]. *C. pubiflora* demonstrates strong antibacterial capabilities against endodontic infections and dental caries, attributed to the active ingredients present in its oleoresin and hydroalcoholic extract [52]. *I. viscosa* effectively inhibits microbial adhesion in the oral cavity through its active components, including 2,5-Dihydroxy-isocostic acid, 2,3-Dihydroxycostic acid, and hydroxy-allylic, dichloromethane-MeOH [58]. Finally, *M. alternifolia* essential oil shows inhibitory effects on bacterial growth associated with dental conditions such as dental caries and periodontitis [90].

In conclusion, the diverse array of botanical sources provides a rich tapestry of compounds, including phenolic acids, tannins, flavonoids, terpenes, and terpenoids, each possessing unique mechanisms of action against oral pathogens. From disrupting bacterial cell walls and inhibiting biofilm formation to targeting cellular membranes and quorum sensing, these natural compounds offer a multifaceted approach to maintaining oral hygiene. Plants like *C. canephora*, *A. indica*, and *O. americanum* showcase potent antimicrobial properties, while essential oils from *E. globulus* and *M. alternifolia* demonstrate effectiveness comparable to synthetic solutions. Harnessing the therapeutic potential of botanical remedies not only enhances oral health but also highlights the significance of biodiversity in promoting overall well-being.

## 5. Scientific Evidence: Clinical and Experimental Studies

Oral diseases can result from bacterial, fungal, or viral infections, as well as from dietary habits and lifestyle factors. Medicinal plants have been used for thousands of years to promote oral hygiene and manage conditions such as dental caries and periodontal diseases. The beneficial effects of these plants are largely attributed to the presence of bioactive compounds that help reduce microbial load in the oral cavity, thereby preventing plaque formation and other oral health issues such as bleeding gums, mouth ulcers, dental caries, gingivitis, and halitosis. Importantly, most oral diseases are preventable and can be effectively managed in their early stages [36].

Natural products derived from medicinal plants play a significant role in maintaining oral health. These products are commonly consumed as extracts or essential oils, or in more traditional forms, such as chewing raw plant parts including leaves, stems, or fruits [26]. The antimicrobial activity of medicinal plants has been widely studied, typically using standardized microbiological assays such as agar disk diffusion (ADD) and micro-broth dilution methods to determine the minimum inhibitory concentration (MIC) of the tested compounds [98]. Additional methods include determination of the minimum bactericidal/fungicidal concentration (MBC/MFC) and the time-kill kinetic assays [99,100]. These methodologies assess either the inhibition of microbial growth or the induction of microbial cell death.

The antimicrobial properties of various medicinal plant species have been tested against Gram-negative anaerobes such as *P. gingivalis*, *Prevotella intermedia*, and *F. nucleatum*—organisms commonly associated with periodontal disease. Although some studies have also targeted cariogenic species and other pathogens involved in dental conditions, most of these investigations have relied on monoaxenic cultures under laboratory conditions. To date, there is limited evidence regarding the effects of these plant-derived compounds in human populations, and only a small number of these species have been evaluated in clinical trials. This highlights the urgent need for more clinical research to validate the antimicrobial efficacy of phytochemicals, which represents a promising alternative to conventional antibiotics for the prevention and treatment of oral infections.

Among the medicinal plants whose anticaries effects have been evaluated in clinical trials are *A. vera*, *A. sativum*, *Rosmarinus officinalis*, *Ocimum sanctum*, *Salvadora persica*, *C. sinensis*, *Vitis vinifera*, *Punica granatum*, *P. guajava*, *T. chebula*, *Lentinula edodes*, *Cichorium intybus*, *A. indica*, and *Streblus asper*. The anticaries properties of these plants—whether in the form of herbal extracts or essential oils derived from various parts of the plant (leaves, fruits, flowers, bark, or roots)—have been tested individually or in combination. These compounds have been incorporated into different delivery formats, including mouthwashes, toothpastes, chewing gums, herbal teas, and juices. Table 5 presents a summary of key clinical studies in which the anticaries efficacy of these species has been demonstrated.

The use of phytochemical compounds in dental applications primarily involves ethanolic or aqueous plant extracts, which are incorporated into mouthwashes and toothpastes. These extracts are derived from various plant parts, including leaves, roots, and seeds. However, the extraction protocols employed differ significantly across research groups, and there is currently no standardized method for the preparation of these extracts. Furthermore, clinical studies assessing the effectiveness of phytochemicals have employed diverse methodologies—including bacterial counts, plaque reduction, and antioxidant activity assays—to evaluate their biological effects. It is noteworthy that most of these studies have short durations, typically ranging from two weeks to two months. Such timeframes are often insufficient to accurately assess the long-term efficacy of plant extracts in preventing or reversing dental caries. Consequently, many clinical studies limit their findings to antimicrobial effects without providing mechanistic insights into their broader therapeutic actions.

Although a substantial amount of information is available regarding the anticaries properties of medicinal plants, most of it is derived from in vitro studies focused on antibacterial activity. Only a limited number of investigations have extended this research to in vivo or clinical settings.

Given that dental plaque is a key risk factor in the development of caries—and that it constitutes a complex biofilm formed by diverse microbial communities—it is essential to conduct in vivo studies that evaluate the effects of phytochemicals, either individually or in combination, on these complex microbial ecosystems rather than on isolated axenic strains alone. Recent research has highlighted that the biological activity of phytochemicals is influenced by the method of extraction used, underscoring the need for methodological consistency and optimization in future studies [118].

## 6. Effectiveness and Safety of Plant-Based Therapies

### 6.1. Analysis of the Efficacy of Natural Therapies

**Figure 1:**
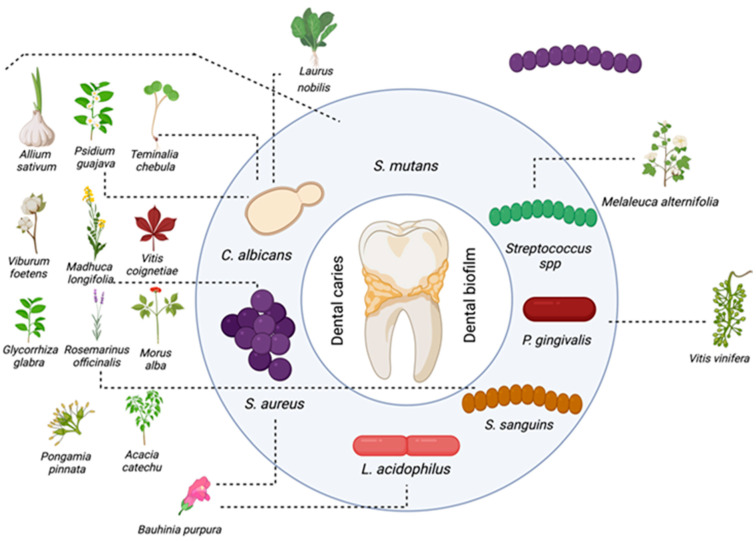
Natural products against dental caries.

Researchers have increasingly focused on the potential of natural products for the prevention and treatment of dental caries (Figure 1). Among these, polyphenols have emerged as some of the most potent bioactive compounds. Nevertheless, the preventive efficacy of natural products remains generally lower than that of conventional agents, such as antibiotics and fluoride. Additionally, the mechanisms of action of many natural compounds are still not fully understood. Greater efforts are needed to identify promising plant-derived agents, elucidate their mechanisms, and refine their formulation to improve their effectiveness against dental caries [119].

In 2019, Ancuceanu et al. [38] reviewed over 56 clinical studies examining the use of herbal products in the prevention of dental caries. Most of these studies focused on assessing the antimicrobial activity of the products, particularly their effects on *S. mutans* and, to a lesser extent, other bacterial or fungal species. Some investigations also evaluated other parameters, such as dental plaque, pH levels, salivary flow, ionic concentrations, and the activity of human salivary amylase. Mouthwash was the most commonly used dosage form, while dentifrices and other delivery methods were less frequently utilized. Certain studies targeted only pediatric populations, and many interventions lasted less than one week, with approximately one-third employing a single application. Chlorhexidine was used as a positive control in nearly one-third of the studies, while 17.86% did not include any control group. Notably, methodological weaknesses and potential biases were reported in four out of five studies, complicating the interpretation of results. Among the most frequently studied species were *C. sinensis*, *T. chebula*, and *Glycyrrhiza uralensis*. Although 85.71% of the trials reported positive outcomes, the overall methodological quality of the studies was considered poor [38].

A subsequent review conducted in 2021 evaluated 47 clinical studies on the efficacy of plant extracts or pharmaceutical formulations containing standardized plant-derived compounds for conditions related to dental caries. This analysis included both microbiological and clinical outcomes. Similar to the findings reported by Ancuceanu, only 15 of the 47 studies presented microbial data, with most showing reductions in total bacterial counts and *S. mutans* levels. In terms of clinical outcomes, many studies reported decreases in plaque and gingival indices [38]. While natural treatments were generally associated with minimal adverse effects, some common complaints included an unpleasant or bitter taste, odor, tooth staining, and a mild burning sensation [120].

López-Villarreal et al. (2022) [121] reported that ethanol extracts of *A. vera, Equisetum arvense, Mimosa tenuiflora, Lippia graveolens*, and *S. aromaticum* demonstrated antibacterial activity against *S. mutans* and *S. sobrinus*. These extracts also exhibited antioxidant and anti-inflammatory effects, as evidenced by modulation of interleukins IL-10, IL-1β, and TNF-α [121].

Table 6 presents widely used plant extracts with demonstrated efficacy against cariogenic microorganisms, along with their corresponding minimum inhibitory concentrations (MICs) [63,122,123]. However, it is important to note that MIC values alone do not guarantee that these extracts will reverse the cariogenic process or serve as replacements for conventional therapies currently recommended by dental professionals.

### 6.2. Discussion and Comparison with Traditional Approaches

The conventional approach to treating dental caries typically falls into three main categories: reparative strategies, therapeutic materials, and behavioral modifications. Reparative strategies, which are well established in clinical dentistry, include techniques such as remineralization and restorative procedures. Following this, root canal disinfection and sealing are performed to eliminate sources of infection. This process involves the removal of necrotic pulp tissue and the reduction in root canal bacterial load through the use of antimicrobial irrigants, either sequentially or in combination.

Sodium hypochlorite (NaOCl) is the most widely used irrigation solution in endodontic therapy due to its broad-spectrum antimicrobial activity and its ability to dissolve necrotic tissue. However, its use is associated with notable disadvantages, including an unpleasant odor and taste, as well as cytotoxic effects on surrounding tissues. Chlorhexidine (CHX), a cationic bisbiguanide, is extensively employed as a final irrigant in endodontics. It exhibits antimicrobial properties and reduces bacterial adhesion to dentin, with broad-spectrum activity at a 2% concentration.

Alexidine (ALX), a bisbiguanide compound structurally similar to CHX, is also used as an antiseptic agent in mouthwashes. Its structural properties allow for hydrophobic interactions with lipid membranes and electrostatic adherence to bacterial cell surfaces. Compared to CHX, ALX demonstrates faster bactericidal activity and increased bacterial membrane permeabilization. Furthermore, unlike CHX, ALX does not form an insoluble para-chloroaniline (PCA) precipitate when combined with NaOCl.

Despite the effectiveness of these agents, biofilms are often highly resistant to disruption. It may take up to two years of consistent antimicrobial use to alter the biofilm composition and significantly reduce caries risk. Moreover, patients with low biofilm loads but other risk factors may not benefit substantially from antimicrobial therapies. Therefore, developing a personalized and effective treatment plan for each patient remains critical [124,125].

Among available antimicrobial agents, CHX is the most extensively studied for its efficacy in controlling cariogenic bacteria. It has been shown to reduce the population of specific microorganisms, particularly *S. mutans*, which is highly susceptible to CHX. Various CHX formulations are commercially available, including dentifrices (0.4%), solutions (0.12% and 0.2%), gels (1%), and varnishes (1%, 10%, 20%, and 35%) [126]. Table 7 presents the minimum inhibitory concentrations (MICs) and minimum bactericidal concentrations (MBCs) of commonly used antimicrobial irrigants traditionally applied against *S. mutans*.

Some studies have demonstrated that antimicrobial agents at sub-minimum inhibitory concentrations (sub-MICs) can effectively inhibit biofilm formation. However, conflicting evidence suggests that sub-MIC levels may, conversely, stimulate biofilm development. The presence of sub-MICs can act as a stress signal for *S. mutans*, triggering adaptive responses through modulation of gene expression, often mediated by quorum sensing mechanisms. A 2012 study reported a significant upregulation of *S. mutans* genes associated with biofilm formation in the presence of sub-MIC levels of antimicrobial agents, suggesting that such concentrations may inadvertently promote biofilm development. Consequently, the use of sub-therapeutic doses of anticaries agents may not be effective and could potentially undermine efforts to prevent or control of dental caries [127].

The exploration of natural products as alternative therapeutic agents against dental caries has gained significant interest, with polyphenols in particular emerging as bioactive compounds of notable potential. However, comparative analyses indicate that, while promising, natural products may not yet achieve the same level of preventive efficacy as conventional chemical agents such as synthetic antimicrobials or fluoride. This limitation is largely due to the limited understanding of the mechanisms of action of most phytochemicals, highlighting the urgent need for intensified research efforts to identify, isolate, and characterize the most effective natural agents.

Despite the therapeutic promise of herbal formulations, the methodological weaknesses observed in many clinical studies pose significant challenges in drawing firm conclusions regarding their efficacy. Variability in study parameters—including participant age, treatment duration, and frequency of application—further complicates the interpretation of results. These inconsistencies underscore the need for standardized protocols and robust study designs in future research focused on natural products for dental caries prevention.

In addition to methodological concerns, several adverse effects commonly associated with herbal preparations—such as unpleasant taste or odor, staining, and mild mucosal irritation—underscore the need for improved formulations and more rigorous evaluation protocols. While the information presented in this review is valuable, it is important to note that MIC values alone are insufficient to ensure a reversal of the cariogenic process or to replace conventional treatments. The successful translation of in vitro findings into clinical practice remains a complex challenge that requires further exploration.

Moreover, the observed enhancement of *S. mutans* biofilm formation in response to sub-MIC levels of antimicrobial agents raises concerns about the potential efficacy of low-dose herbal formulations, especially those with poorly understood mechanisms of action. Although traditional agents offer proven benefits, their limitations—ranging from cytotoxicity to antimicrobial resistance—emphasize the continuing need to develop safer, more effective alternatives.

In conclusion, the prevention of dental caries is a multifaceted challenge that requires comprehensive research, well-designed clinical trials, and the development of standardized, scientifically validated herbal formulations. Bridging the gap between promising laboratory results and effective clinical applications will be essential to establish the therapeutic viability of natural products in modern dental care.

## 7. Plant-Based Nanoparticles and Coatings with Antibacterial Activity for Caries Treatment

### 7.1. Nanoparticles

Nanomaterials are materials with a size range between 1 and 100 nm. The morphology and size distribution of nanoparticles (NPs) are key factors for their inventive bio-applications including in oral care products [128]. Metal nanoparticles that have received special attention in recent years are silver nanoparticles (AgNPs), which have been demonstrated to be effective antimicrobial components in prosthetic materials, adhesives and implants, to promote caries arrestment, to prevent biofilm formation and for osteogenic induction [129]. Other silver-based compounds have also been successfully applied in dentistry. For example, the linear complex [Ag(NH_3_)_2_]^+^F, which was recently cleared for caries arrestment in the US, has been used in Japan for more than 80 years. It is now possible to produce AgNPs with controlled size and morphology, high homogeneity (i.e., low polydispersity index), and specific target functions (i.e., functionalized with molecular capping agents, from small hydrophilic and hydrophobic chemical groups to large biomacromolecules, such as proteins). AgNPs can access different sites in the oral cavity; in such a way, they can currently be conceived as multifunctional building blocks for dental materials and dentistry protocols [129]. Bacteria in the oral cavity are organized in biofilms, which confer better conditions for growth, immunological evasion, and resistance to antibiotics. Preparing NPs must consider biofilm architecture. The NPs’ properties may affect their efficiency and interfere with their mechanism of action. Some important aspects to mention in this area include the diffusion of nanoparticles in biofilms, which exhibits an inverse relationship between effectiveness and size; nanoparticles over 50 nm are not able to penetrate the biofilm due to the relative self-diffusion coefficients in the biofilm, and this decreases exponentially with the square of the nanoparticle diameter [129]. Plant extracts are widely used for reasons such as vast and accessible reserves, large distribution, safe handling, availability of a wide range of metabolites with strong reducing potentials, and minimal waste and energy costs [130].

Plaque biofilm formation is one of the causes of dental diseases. AgNPs have been incorporated into some dental biomaterials for reducing biofilm formation due to their antibacterial activity. AgNPs exhibited stronger inhibition of Gram-negative organisms, which were primarily responsible for periodontal infection [131].

Bioactive AgNPs have been synthesized using various plant extracts from *Punica granatum*, *Justica glauca* leaf, and *Plectranthus ambionicus.* It has been investigated that they have antimicrobial activity against oral microorganisms such as *S. mutans*, *C. albicans*, *L. acidophilus*, and *E. faecalis* [128].

Pomegranate is characterized by high phenolic contents—punicalagin, punicalin, ellagitannins, gallic acid, ellagic acid and anthocyanins—that exhibit favorable properties against various inflammatory disorders. *P. granatum* nanoparticles associated with calcium glycerophosphate (CaGP) have been synthesized, characterized, and evaluated for their antimicrobial activity against planktonic cells and biofilms of *S. mutans* and *C. albicans* [130].

Green synthesis of AgNPs employing the leaf extract of *Justicia glauca* was developed and tested for antimicrobial activity against dental caries and periodontal disease-causing microorganisms such as *S. mutans*, *S. aureus*, *L. acidophilus*, *Micrococcus luteus*, *B. subtilis*, *E. coli*, *Pseudomonas aeruginosa* and *C. albicans* [132]. Extracts of *Plectrantus ambionicus* were incorporated into AgNPs against *E. faecalis* and *C. Albicans.* The result suggests that silver nanoparticles can be used as inhibitors against *E. faecalis* and *C. albicans*, making them applicable in anti-microbial control systems [133].

**Figure 2:**
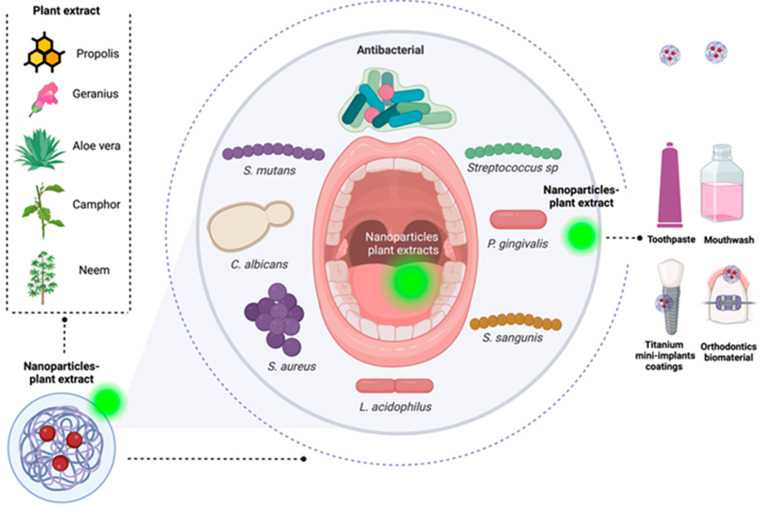
Plant-based nanoparticles (NPs) used in buccal treatments.

Different treatments have been developed against the microorganisms responsible for diseases in nanoparticle-based buccal membranes using natural products and plant extracts (Figure 2). Nanomaterials can also deliver oral fluids or pharmaceuticals, treating oral cancers and providing a high level of oral healthcare. These are also found in toothpaste, mouthwash, and other dental care products [134]. For example, propolis, a natural beehive product, is a complex resinous material inhibiting *S. mutans* growth and adhering to tooth surfaces. Specially prepared dental varnishes containing propolis, Miswak, and chitosan nanoparticles (CS-NPs) with or without sodium fluoride (NaF) were assessed for antibacterial effect against *S. mutans* using a disk diffusion test. Fluoride-containing Miswak varnish (MF) and CSF-NPs varnish inhibited demineralization significantly better than all experimental varnishes, especially during the first 2 days, though CSF-NPs varnish had a low fluoride concentration, probably due to better availability of fluoride ions and the smaller size of nanoparticles. Incorporating natural products with fluoride into dental varnishes can be an effective approach for caries prevention, especially Miswak and propolis when financial resources are limited [135].

In vitro studies demonstrate that AgNPs have a strong antibacterial effect when coupled with dental materials, including acrylic resins, nanocomposites, adhesives, resin comonomers, intracanal medications, and implant coatings [134]. Dentures, which are usually made of polymethyl methacrylate (PMMA) acrylic resin, have a rough internal surface, along with other factors (such as poor hygiene and HIV infection), leading to the development of denture stomatitis. *Candida* species colonize denture surfaces, generating a biofilm that can induce the development of denture stomatitis [136]. Acosta-Torres et al. (2012) created PMMA with 1 μg/mL AgNPs and compared it to PMMA that had not been changed. PMMA AgNP specimens revealed reduced *C. albicans* adhesion more than PMMA [137].

Because of their durability and exceptional strength, ZrNPs are frequently utilized in dentistry due to zirconia’s superior chemical stability, biocompatibility, adequate fracture resistance, and flexural strength. Also, due to decreased porosity and increased thickness, zirconia nanoparticles improved the coating’s abrasion behavior. Both histological and cytological studies have shown that the level of toxicity is moderately low [138]. Compared to traditional organic antibacterial agents, it is superior in terms of reliability, resilience, and heat resistance against bacterial pathogens apart from *S. aureus* and *E. coli*, in addition to antifungal effectiveness against *C. albicans* and *Aspergillus Niger*. Due to the excessively straightforward, nontoxic, quick, ecologically safe, cost-effective, and simple one-step method to manufacture NPs, using plant material to synthesize zirconium dioxide nanoparticles generated great enthusiasm. That biomolecule includes tannins, sugar, steroid, enzymes, phenols, amino acids, flavonoids, and sugar, typically obtained from natural extracts and for significant overall medical purposes. The plants also include diverse combinations of biomolecules that aid in the stability of ZrO_2_ nanoparticles [139]. Creating NPs using plant extracts is non-toxic, rendering plants the best choice. Plant extracts like geranium, *A. vera*, sun-dried *Cinnamomum camphor*, *A. indica*, etc., can be incrusted in silver and gold NPs [140].

Extensive research has been performed on dental implant applications regarding the sustainable production of plant nanoparticles. Compared to traditional organic antibacterial agents, it is superior in terms of reliability, resilience, and heat resistance against bacterial pathogens apart from *S. aureus* and *E. coli*, in addition to antifungal effectiveness against *C. albicans* and *Aspergillus niger* [138].

Wassel, M. O. and Khattab, M. A. (2017) [135] investigated the application of propolis, Miswak, and chitosan nanoparticles (CS-NPs) with or without sodium fluoride (NaF) for antibacterial effect against *S.* mutans. While adding fluoride to the tested natural products increased the antibacterial activity more than each natural product alone, this difference was not significant compared to varnishes containing only the corresponding natural product but was significant compared to fluoride varnish, indicating an additive effect with fluoride, which supports the limited antibacterial activity of fluoride.

Silver NPs coated onto metal are known to reduce the colonization of bacteria; thus, coating mini-implants will reduce the risk of peri-implantitis and implant failure. Mini-implants coated with silver nanoparticles were tested for antimicrobial properties against *S. mutans*, *S. aureus*, *Lactobacillus* and *C. albicans*. A considerable zone of inhibition was also formed after 24 to 48 h of incubation of silver nanoparticles in *C. albicans*. Chitosan–silver NPs have strong antibacterial activity against *S. aureus*, *S. mutans*, and *Lactobacillus* species [141]. Sondi and Salopek-Sondi demonstrated that AgNPs have excellent antibacterial activity against *E. coli* [142]. In the presence of 105 colony-forming units (CFU) of *E. coli*, 10 μg/cm^3^ of AgNPs inhibited bacterial growth by 70% [130].

Recent studies have explored the nanoencapsulation of diverse natural products into NPs to enhance their stability, bioavailability, and efficacy against cariogenic microorganisms. These nanoformulations represent a promising alternative approach for the prevention and treatment of dental caries, as summarized in Table 8.

Previous research investigated the mechanical characteristics and biofilm formation of AgNPs inserted into composite resin in a study published in 2013. AgNPs were added at concentrations of 0.028 and 0.042. When the AgNP concentration was 0.042, however, *S. mutans* colony-forming units were reduced by 75% [150].

Rodrigues et al. (2020) demonstrated that Ag-SiO2 NPs of *C. sinensis* (green tea) exhibited strong antibacterial activity against *S. mutans*, with a 600 g/mL MIC and biofilm inhibition of around 44% and 11 nm (spherical shape) [145]. When compared to CAgNPs, MAgNPs were found to have better antibacterial action. At doses of 3 g/mL and 50 g/mL, respectively, MAgNPs and CAgNPs inhibited *S. mutans* biofilm formation by 99 and 94 percent, respectively [151], with an NP size of 10–30 (irregular shape) [152]. The combination of *Ficus benghalensis* and AgO_2_NPs has good antibacterial activity against *S. mutans* and *Lactobacilli* sp., two dental pathogens with an NP size of 42.7 nm.

### 7.2. Coatings

Titanium mini-implants when coated with silver NPs have excellent antimicrobial properties and, hence, can be used as biomaterial in orthodontics, but further tests are needed to evaluate the coating during and after placement [141]. In orthodontic patients, there is a documented increase in *Streptococcus* and *Lactobacillus* [153]. Silver nanoparticles have previously been coated onto hexagon dental implants and have shown positive antimicrobial activity against *S. aureus* [154].

Surface modification with Ag NPs is highly recommended for dental implants because of their ability to promote osteogenesis and soft-tissue integration. To provide synergistic antibacterial (*S. epidermidis*, *S. mutans*, and *E. coli*) and osteogenic (human osteoblast-like cells, SAOS-2) properties, AgNPs have been deposited on Ti implants via anodic spark deposition [155]. A study shows that 30 nm sized AgNPs doped with Ti alloy showed better antimicrobial and biocompatibility [156]. As a surface modifier of dental implants, AgNP-coated Ti showed better antimicrobial activity against *S. mutans* and *P. gingivalis* [157]. As shown in Table 9, various oral coatings have demonstrated effectiveness against cariogenic microorganisms, highlighting their potential as preventive agents in dental care.

## 8. Future Perspectives and Challenges

Medicinal plants have shown promising potential as treatments for dental caries [15]. While many studies highlight the beneficial effects of herbal medicines in dentistry and suggest that these products can serve as alternatives to conventional drugs—often without the associated side effects—there remains a significant lack of evidence regarding their safety, biocompatibility, and clinical efficacy. Most available research has been conducted in in vitro or preclinical settings, underscoring the urgent need for increased research efforts and funding directed toward clinical trials that assess the efficacy, safety, cost-effectiveness, and pharmacological characterization of these natural compounds.

A major challenge in advancing phytotherapeutic agents into dental practice is the limited information about their specific effects on oral tissues, mechanisms of action, and potential adverse effects [26]. This is particularly relevant in the context of global health, as alternative and herbal therapies may be especially valuable in low-resource settings where access to conventional dental care is limited [22].

The medicinal use of plants continues to be a widespread practice among human populations [15], and they remain a core component of ethnomedicine [37]. Herbal medicines have been shown to exhibit a wide array of biological activities, including antimicrobial, antioxidant, and anti-inflammatory effects, at both oral and systemic levels [20]. Given the growing need for new therapeutic alternatives in dentistry, plant-derived compounds represent a highly relevant and attractive option due to their efficacy and typically low toxicity profiles [22].

One of the most important biological properties of phytocompounds is their antibacterial activity, which is instrumental in addressing common oral health problems. A key advantage of medicinal plants lies in their complex mechanisms of antibacterial action, which may reduce the risk of bacterial resistance development [15]. Moreover, the synergistic interactions among multiple phytochemicals in a single plant extract often enhance therapeutic outcomes beyond what individual compounds can achieve.

Nevertheless, phytochemicals should also be studied in combination with other plant-derived compounds as well as with conventional drugs currently used in dental therapy, to better understand potential synergistic or antagonistic effects [26]. Despite their many benefits, some natural compounds may cause serious adverse reactions and thus require careful evaluation. For safe therapeutic use, the botanical origin of the medicinal plant must be clearly identified, and in the case of isolated compounds, appropriate qualitative and quantitative control must be ensured.

Future research should also target developed countries, where rising rates of bacterial and viral resistance pose a significant public health challenge and where dental diseases remain highly prevalent. Large-scale, population-based studies are necessary to validate the clinical effects of herbal and phytotherapeutic agents, especially given the mixed results reported in existing trials [20].

In summary, alternative and herbal therapies offer promising solutions for improving global oral health, especially in underserved populations [22]. These scientific findings may serve as a foundation for future drug discovery and the development of novel antimicrobial agents [37].

## 9. Conclusions and Future Perspectives

The growing interest in medicinal plants as alternatives for the prevention and treatment of dental caries highlights their potential as effective antimicrobial agents. Numerous plant-derived compounds, including polyphenols, alkaloids, and essential oils, have demonstrated significant antibacterial activity against a broad spectrum of microorganisms associated with oral diseases. In addition to their direct antimicrobial effects, these compounds also exhibit anti-inflammatory and antioxidant properties that contribute to overall oral health maintenance.

Despite these promising findings, the majority of existing studies are limited to in vitro and in vivo models, with a notable lack of large-scale, randomized clinical trials to assess their long-term safety and therapeutic efficacy in humans.

To advance the integration of plant-based therapies into conventional dental care, future research should aim to standardize extraction methods, improve bioavailability, and determine optimal dosages. The development of novel delivery systems—such as nanoparticle-based formulations—may enhance the stability and therapeutic potential of these phytochemicals. Furthermore, interdisciplinary collaboration among pharmacologists, dentists, and biotechnologists will be essential for translating preclinical data into viable clinical applications. Addressing these challenges could position medicinal plants as a cornerstone in the next generation of preventive and therapeutic strategies for oral health.

## 10. Limitations

Although a considerable number of studies have demonstrated the anticaries activity of various phytochemicals, most of this evidence is derived from in vitro antibacterial assays, with limited validation through clinical studies. There remains a substantial gap in the literature concerning randomized clinical trials evaluating the effectiveness and safety of these natural products in real-world scenarios.

This review only considered publications from 2004 to 2025 and was focused on studies published in scientific journals that featured clinical research. While information from different types of clinical trials was considered—including studies involving children, adults, and elderly populations—many did not provide detailed demographic or methodological information about the study population.

Future work should aim to classify clinical evidence based on variables such as the geographic origin of the plants, characteristics of the study population, plant part utilized, type of extract and extraction method, formulation and route of administration, and dosage and treatment duration. A more systematic approach to categorizing these variables would improve the comparability of results and support the development of standardized, evidence-based applications of medicinal plants in dentistry.

## Figures and Tables

**Table 1:** Application of medicinal plants against dental pathogens in vitro assays.

Species	In Vitro	Reference
*Allium sativum*	Antibacterial effect on multi-drug-resistant species of *Streptococcus mutans,* antitumor and antiproliferative effect on human oral squamous cancer cells	[15]
*Cotinus coggygria*	Potential against dental calculus	[29]
*Propolis*	Antimicrobial against *S. mutans* and anticandidal activities	[15]
*Cocos nucifera*	Antimicrobial activity Antifungal activity	[15]
*Inula viscosa*	Antibacterial activity against cariogenic bacteria	[27]
*Carica papaya*	Removal of dental caries	[15]
*Cistus incanus*	Antibacterial effect against *S. mutans*, prevention of bacterial adhesion, inhibition of glucosyltransferase (GTF)	[27]
A variety of plant species	Anticariogenic activity and antibiofilm formation of a variety of polyphenols	[30]
*Glycyrrhiza glabra*	Antibacterial effects on both Gram-positive and -negative bacterial species. Antibacterial effects on the species *Porphyromonas gingivalis.* Antifungal effects on *Candida albicans*	[15]
*Hypericum perforatum*	Antibacterial effect against *Aggregatibacter actinomycetemcomitans* and *P. gingivalis*	[15]
*Mentha piperita* *Thymus vulgaris*	Antibacterial effects on *S. mutans*, *C. albicans* and *A. actinomycetemcomitans* Antimicrobial effects on *S. mutans*	[15]
*Cinnamomum zeylanicum*	Antimicrobial effects on *S. mutans* and *Lactobacillus acidophilus*	[31]
*Aloe vera*	Bactericidal effects against cariogenica and periodontopatogenic bacterias	[15]
Medicinal Plant Extracts of Cinnamon, Turmeric, Ginger, Clove and Black seed	Antimicrobial effects on *S. mutans* and *L. acidophilus*	[32]
*Melaleuca alternifolia* *Syzygium aromaticum*	Antimicrobial activity Antimicrobial effects on *S. mutans* and *L. acidophilus*	[15,31]
*Peperomia* *pellucida* (EOs) *Piper marginatum* (EOs) *Piper callosum* (EOs)	Antimicrobial effects against *S. mutans*, *Streptococcus mitis*, *Streptococcus sanguinis*, *Streptococcus salivarius*, *S. sobrinus*, *Enterococcus faecalis*, and *Lactobacillus casei*	[33]
*Nigella sativa*	Antimicrobial effects on *S. mutans* and *L. acidophilus*	[31]
*Citrus aurantifolia*	Antimicrobial effects on *S. mutans*	[15]
*Camelia sinensis*	Antimicrobial effects against *S. mutans, P. gingivalis* and *Prevotella nigrescens*	[15]
*Vitis vinifera* (Grape seed extract)	Antimicrobial effects against *S. mutans*	[15]
*Curcuma longa*	Antimicrobial effects on *S. mutans*	[15]
*Zingiber officinale*	Antimicrobial effects on *S. mutans* and *L. acidophilus*	[31]
*Verbascum speciosum*	Anticariogenic activity against *S. mutans* and *Streptococcus sobrinus*	[34]
*Morinda Oleifera*	Antimicrobial effects on *S. mutans*	[35]

**Table 2:** Formulations based on medicinal plants for the treatment of caries in vivo assays.

Medicinal Plants	Formulation	Year
*Camellia sinensis* *Terminalia chebula* *Glycyrrhiza uralensis*	Mouthwash Toothpastes	[38]
*Terminalia chebula* *Psidium guajava* *Azadirachta indica* *Pongamia pinnata* *Syzygium aromaticum* *Mentha piperita*	Mouthwash	[34]
*Syzygium aromaticum* *Dennettia tripetala* *Jatropha curcas*	Toothpastes	[13]
*Copaifera langsdorffii*	Dental varnish	[28]
*Teucrium polium*	Mouthwash	[35]
*Salvadora persica* *Asparagus racemosus* *Streblus asper* *Rosamarinus officinalis*	Toothpastes	[36]
*Piper crocatum*	Mouthwash	[37]
*Miswak*	Toothpastes	[23]

**Table 3:** Medicinal plants/parts against oral microorganisms.

Plant Specie	Family	Part Used	Microorganism	Reference
*Acacia nilotica*	*Fabaceae*	Leaves, stem, bark	*S. mutans*, *Streptococcus sobrinus*, *Porphyromonas gingivalis*	[41,42]
*Allium sativum*	*Amaryllidaceae*	Bulb of the plant	*Enerecoccus faecalis*, *Stapylococcus aureus*, *Escherichia coli*	[43]
*Asparagus racemosus*	*Asparagaceae*	Aerial parts	*S. mutans*, *L. acidophilus*	[18]
*Azadirachta indica*	*Meliaceae*	Bark, leaves, seeds	*S. mutans*, *Sreptococcus salivarius*, *Streptococcus sanguis* and *S. mitis*	[44,45]
*Berberis vulgaris*	*Berberidaceae*	Roots	*S. mutans*, *S. sobrinus*, *S. sanguinis*, *S. salivaris*, *Lactobacillus rhamnosus*	[46]
*Camellia sinensis*	*Theaceae*	Leaf buds, stems	*S. mutans*, *L. acidophilus*	[47,48]
*Citrus sinensis*	*Rutaceae*	Peel, leaves, wood of the tree	*S. mutans*, *L. acidophilus*	[49]
*Cichorium intybus*	*Asteraceae*	Aerial parts	*S. aureus*, *E. coli*, *Salmonella typhi*, *Prevotella intermedia*, *S. mutans*	[50]
*Coffee canephora*	*Rubiaceae*	Beans, stems leaves	*P. gingivalis*	[51]
*Copaifera pubiflora*	*Fabaceae*	Oleoresin, bark	*P. gingivalis Aggregatibacter actinomycetemcomitans*	[52]
*Coriandrum sativum*	*Apiaceae*	Seeds, leaves	*S. mutans*, *C. albicans*	[53]
*Embelia ribes*	*Primulaceae*	Stem, bark	*Bacillus subtilis*, *S. mutans*, *S. sanguinis*	[36,54]
*Eucalyptus globulus*	*Myrtaceae*	Leaves	*S. mutans, E. faecalis*	[55,56]
*Eugenia caryophyllata*	*Myrtaceae*	Gall	*S. mutans*, *L. acidophilus*	[31,57]
*Inula viscosa*	*Asteraceae*	Leaves, stem	*P. gingivalis*, *S. sobrinus*	[58]
*Juglans regia*	*Juglandaceae*	Bark	*P. gingivalis* *S. salivarius*, *S. mutans*, *S. aureus*, *S. sanguis*	[59,60]
*Lippia sidoides*	*Verbenaceae*	Leaves, aerial parts, flowers	*S. mutans*	[53,61]
*Melaleuca alternifolia*	*Myrtaceae*	Leaves	*P. gingivalis*, *Porphyromonas endodontalis*, *A. actinomycetemcomitans*	[62]
*Nigella sativa*	*Ranunculaceae*	Seeds	*S. mutans* *P. intermedia*, *P. gingivalis*, *A. actinomycetemcomitans*	[63,64]
*Ocimum americanum*	*Lamiaceae*	Leaves	*S. mutans* *L. casei*, *S. sanguinis*	[65]
*Ocimum sanctum*	*Lamiaceae* *Labiatae*	Leaves, stem, flower, root, seeds	*S. mutans*, *S. sanguinis* *A. actinomycetemcomitans*	[66]
*Psidium guajava*	*Myrtaceae*	Bark, leaf, stem,	*P. gingivalis*, *P. intermedia*, *S. aureus*, *E. coli*	[67]
*Quercus infectoria*	*Fagaceae*	Galls	*S. mutans*	[68]
*Rosmarinus officinalis*	*Lamiaceae*	Leaves, aerial parts	*S. sanguinus* *S. mutans* *S. sobrinus*	[69,70]
*Salvadora persica*	*Salvadoraceae*	Roots, branches	*S. mutans*, *S. aureus* and *Streptococcus* sp. *isolates*	[71,72,73]
*Streblus asper*	*Moraceae*	Leaves, steam bark, aerial parts	*S. mutans*, *P. gingivalis A. actinomycetemcomitans*	[74]
*Terminalia chebula*	*Combretaceae*	Fruit	*A. actinomycetemcomitans*, dental plaque bacteria, *S. mutans*	[75]
*Thymus vulgaris*	*Lamiaceae*	Leaves	*S. mutans* *C. albicans*	[76]
*Vaccinium macrocarpon*	*Ericaceae*	Fruit	*S. mutans*, *S. sobrinus*, *P. gingivalis, Fusobacterium nucleatum*, *S. mutans*, *Actinomyces naeslundii*	[77]
*Vitis vinifera*	*Vitaceae*	Seeds, leaves	*P. gingivalis*	[78]

**Table 4:** Plant compounds and their bioactivity against cariogenic microorganisms.

Plant Species	Extracts/Key Compounds	Biological Activity	References
*Acacia nilotica*	Stem and bark extracts contain quercetin, luteolin, saponins, anthraquinones, amino acids, fatty acids, diverse carbohydrates, and polyphenols like chebulinic acid alongside tannin, gallic acid, catechin, epigallocatechin-7-gallate, catechin derivatives, ellagic acid, kaempferol and quercetin.	*A. nilotica* are used as antibacterial agents (*S. sobrinus and P. gingivalis*) targeting oral pathogens and are effective in managing dental plaque. Additionally, they can inhibit GTF and its antioxidant properties, with a focus on promoting oral hygiene, by decreasing bacteria counts and controlling dental plaque. It is used as a chewing stick, mouthwash, or gum.	[41,79,80]
*Allium sativum*	Tannins, flavonoids, and alkaloids (alliin, methiin, sodium acetate).	The substances exhibit antibacterial, antifungal, and antiviral characteristics, making them suitable for treating dental cavities. They are formulated into gels, gums, toothpaste, and pharmaceutical strips for oral care applications.	[81,82]
*Asparagus racemosus*	Borneol, myrtanol, pinocarveol, 2-ethylhexanol, perillaldehyde	Antibacterial properties against *S.mutans* and *L. acidophilus*	[18]
*Azaridachta indica*	Extracts from bark, leaves, and seeds display antifungal, anti-ulcer, and antinociceptive properties. Contains approximately 300 structurally distinct constituents, with the majority being limonoids. The composition includes hexadecanoic acid, oleic acid, 5,6-dihydro-2,4,6-triethyl-(4H)-1,3,5-dithiazine, methyl oleate, and eudesm-7(11)-en-4-ol, tannins, lignins, flavonoids	Extract from neem demonstrates having efficacy against bacteria such as *S. mutans, S. salivarius, S. sanguis,* and *S. mitis*. A blend of chewing sticks proves advantageous in eliminating organisms responsible for dental caries; chewing these sticks increases saliva secretion, cleansing, and antibacterial, antioxidant, and anti-inflammatory properties, and also it helps to reduce the oxidative stress that comes with periodontal disease. It has also shown antimicrobial properties by destructing bacterial cell membranes which reduces the surface of adhesion of specific bacteria and inhibits bacterial growth. Moreover, a mucoadhesive dental gel infused with *A. indica* exhibits significant benefits by reducing both the plaque index and salivary bacterial count, surpassing the effectiveness of CHX gluconate mouthwash.	[34,83]
*Berberis vulgaris*	Protoberberine, alkaloids, roots	Dental gel. Antibacterial properties, plaque formation index decreasedThe growth of microorganisms is inhibited by berberine.	[84]
*Camellia sinensis*	Epicatechin-3-gallate, epigallocatechin-3-gallate	An effective treatment and preventive measure against periodontal disease, possessing antioxidant, anti-inflammatory, antibacterial, antiviral, and antimutagenic properties. They can potentially be used in the preparation of toothpastes and mouthwashes.	[85]
*Citrus sinensis*	Orange peel extract	Antibacterial properties: numerous studies have revealed that it can treat periodontal disease	[49,60]
*Cichorium intybus*	The extract comprises primarily carvacrol, cinnamic aldehyde, thymol, camphor, linalool, carvone, and terpineol. Additionally, it contains inulin, caffeic acid derivatives like ferulic acid, caftaric acid, chicoric acid, and chlorogenic acid (3-CQA), as well as isochlorogenic acid (3,5-diCQA), dicaffeoyl tartaric acid, sugars, proteins, hydroxycoumarins, flavonoids, terpenoids, sesquiterpene lactones, alkaloids, steroids, oils, volatile compounds, vitamins, and polyynes.	Potential in preventing virulence-linked properties of oral pathogens such as *P. intermedia* and *S. mutans*	[86]
*Coffea canephora*	Phenolic acid, green coffee extract	Green coffee extract’s chlorogenic acid decreased the oral bacteria *S. mutans* in a clinical experiment.	[87,88]
*Copaifera pubiflora*	Oleoresin, hydroalcoholic extract	Antibacterial in endodontic infections and dental caries; antimicrobial properties against *P. gingivalis* and *A. actinomycetemcomitans.*	[52]
*Coriandrum sativum*	The leaves predominantly contain decanal, trans-2-decenal, and 2-decen-1-ol. Fatty acids (AKFAs) were extracted from the endophytic fungus *Arthrographis kalrae,* which was isolated from *Coriandrum sativum* leaves. The leaves also contain linalool, terpenoids, phenolic compounds, and fatty acids. The contents of Fraction Cs4 isolated from the leaves include 1-decanol, thymol, trans-caryophyllene, trans-2-dodecen-1-ol, spathulenol, globulol, and α-cadinol.	The essential oil of *C. sativum* exhibits antifungal activity, significantly reduces the viability of *C. albicans* cells, and demonstrates potential against *S. mutans* biofilms, combating dental caries.	[53]
*Eucalyptus globulus*	The essential oil extracted from leaves contains a total of 27 compounds. The key compounds present in the oil include β-pinene, eucalyptol (1,8-cineole), α-pinene, α-phellandrene, gamma-eudesmol, para-cymene, β-eudesmol, limonene, terpinen-4-ol, and piperitone.	Demonstrates significant antimicrobial efficacy against bacteria commonly present in the oral cavity. The EO notably hindered both the planktonic and biofilm growth of *S. mutans* and *E. faecalis*. In this way, EOs can be used to generate pharmaceutical products for oral health.	[55]
*Eugenia caryophyllata*	The primary constituents present in clove oil are phenylpropanoids, notably eugenol and β-caryophyllene, 2-methoxy-4-(2-propenyl)-, acetate.	Clove oil, renowned for its antiseptic, analgesic, and anesthetic effects, displays robust antimicrobial activity against streptococci, particularly *S. mutans*. Eugenol, a major component of clove oil, showcases potent antimicrobial properties against cariogenic bacteria, specifically targeting *S. mutans*. Moreover, the methanolic extract of clove demonstrates significant antimicrobial efficacy against both *S. mutans* and *L. acidophilus*, suggesting its potential application in minimally invasive and adhesive dentistry practices. Also, clove exhibits sensibility to pathogens and bacteria associated with tooth decay and periodontal diseases, offering antibacterial activity against *P. gingivalis* and *P. intermedia*, alongside antioxidant properties.	[53,57]
*Inula viscosa*	2,5-Dihydroxy-isocostic acid, 2,3-Dihydroxycostic acid, hydroxy-allylic, dichloromethane-MeOH.	Inhibits microbial adhesion in the oral cavity. It has antibacterial activity against *P. gingivalis*, and *S. sobrinus*.	[58,89]
*Juglans regia*	The bark extract contains juglone, terpenoids, alkaloids, steroids, phenols flavonoids, saponins, polyphenols, acid, mono, di, tri acylglycerol, free fatty acids, oleic and linoleic acids, proteins, naphthaquinones, ascorbic acid, sitosterol, and tannins.	Extract from *Juglans regia* with juglone exhibits antibiofilm and growth inhibitory effects against oral pathogen *P. gingivalis*, enhancing oral hygiene. Bark of *J. regia* is side effect-free, beneficial against plaque and caries bacteria, contrasting with harmful mouthwashes and antibiotics. It also reduces *P. gingivalis* growth and has antibiofilm action against *S. sobrinus*, *A. viscosus*, and *S. mutans*, with antiplaque formation properties.	[59]
*Lippia sidoides*	Essential oil rich in thymol, carvacrol, flavonoids.	Potent antibacterial activity against cariogenic bacteria; effective against plaque and gingivitis. Extracts from *L. sidoides* significantly reduce extracellular polysaccharides and bacterial cells in *S. mutans* biofilm, without affecting biofilm thickness. *L. sidoides* essential oil shows strong antibacterial activity and clinical efficacy as a mouthwash, making it promising for combating plaque and gingivitis. It also manages supragingival biofilm, inhibits plaque formation, and possesses antigingivitis properties.	[51,53]
*Melaleuca alternifolia*	The essential oil extracted from leaves and branches contains a variety of compounds including terpinen-4-ol, 1,8-cineole, alpha-terpineol, gamma-terpinene, terpinene, terpinolene, cymene, limonene, pinene, sabinene, viridiflorol, and globulol.	Displayed inhibitory effects on bacterial growth linked with various dental conditions including dental caries, periodontitis, dental plaque, and gingivitis. The bacteria affected include *P. gingivalis*, *P. endodontalis*, and *A. actinomycetemcomitans.*	[90]
*Nigella sativa*	The active extract, especially in nanoemulsion form, is attributed to its key components, including carvacrol, longifolene, ρ-cymene, t-anethole, 4-terpineol, and thymoquinone.	The essential oil exhibits anticarcinogenic, antioxidant, and antimicrobial properties. It has also demonstrates sensitivity against intermedia, *P. gingivalis*, and *A. actinomycetemcomitans*. Its suggests that thymoquinone could be significant in both the treatment and prevention of periodontal diseases.	[64]
*Ocium americanum*	The essential oil of *Ocium* leaf contains medium-chain free fatty acids derivative of lauric acid; it is a type of fatty acid that interacts with MurA enzyme, which is necessary for the formation of the cell wall of cariogenic bacteria.	The essential oil extracted from the leaves of *Ocimum americanum* demonstrates grand antimicrobial activity against cariogenic bacteria, notably *S. mutans*, *S. sanguinis* and *L. casei*, in both planktonic and biofilm cultures, comparable efficacy to CHX solution in reducing bacterial counts. This plant also showed activity against periodontal microorganisms *P. gingivalis, P. intermedia*, and *F. nucleatum*.	[53,65]
*Ocimum sanctum*	The essential oil contains caryophyllene, β-caryophyllene, β-pinene, copaene, and eugenol. Additionally, *O. sanctum* essential oil comprises a diverse range of groups, including monoterpenes hydrocarbons (e.g., α-pinene, camphene), sesquiterpene hydrocarbons (e.g., caryophyllene, Copaene, α-caryophyllene, α-bourbonene, α-cubebene), oxygenated monoterpenes (e.g., caryophyllene oxide), and aromatic compounds (e.g., eugenol, borneol, methyl iso-eugenol).	The ethanol extract displays antibacterial effectiveness against *S. mutans* and has long been utilized for alleviating toothaches and pulpitis traditionally. These components contribute to the antimicrobial attributes of Tulsi, rendering it efficient against bacteria responsible for dental caries. The essential oil of *O. sanctum* exhibits antimicrobial and antifungal properties against oral pathogens associated with dental issues. It is utilized as an ingredient in mouthwash and toothpaste formulations by pharmaceutical companies for treating toothaches and pulpitis. Eugenol, extensively utilized in dentistry, is one of its key compounds.	[66]
*Psidium guajava*	Bark, leaf, stem. Flavonoids: guaijaverin, quercetin, tannins.	Paste and mouthwash. Anti-inflammatory, antioxidant, antimicrobial and wound-healer properties. Inhibits *P. gingivalis* and *P. intermedia* growth, antiplaque formation	[67]
*Quercus infectoria*	The extract is rich in tannins; it also contains gallic acid and ellagic acid.	The extract demonstrates antimicrobial properties, proving effective against the causative agents of both periodontitis and dental caries. The extract significantly reduced the formation of biofilm biomass by *S. mutans*, indicating its potential in combating dental caries. The EO showed activity against various oral pathogens, including *F. nucleatum*, *S. mutans*, *S. salivarius* and *P. gingivalis.*	[68]
*Rosmarinus officinalis*	Essential oil contains terpenoids, flavonoids, phenols, essential oils, borneol, camphor and verbenone.	*R. officianalis* essential oil has antioxidant, antibacterial (*S. sanguinus*, *S. mutans*, *S. sobrinus*), antifungal and antibiofilm properties; this aids in preventing plaque formation, and the reduction in biofilm formation suggests potential application in new anticaries treatment protocols.	[91]
*Salvadora persica*	The mixture comprises oxygenated monoterpenes, sesquiterpene hydrocarbons, and monoterpene with primary constituents including α-caryophellene, 9-epi-(E.)-caryophellene, 1,8-cineole (eucalyptol), and β-pinene. Additionally, it contains chrysin-8-c-β-D-glucopyranoside, ferulic acid, gallic acid, stigmasterol, butylated hydroxytoluene, and benzene (isothiocyanatomethyl). Other components include tannins, vitamin C, potassium, sodium chloride, silica, salvadorine, salvadourea, and saponins, along with fibrous branches.	The compounds exhibit plaque formation inhibition and have a traditional use as toothbrushes, recent studies highlighting their effectiveness in combating gingivitis and enhancing oral hygiene. They significantly contribute to the antibacterial activity observed in *Salvadora persica* extract against *S. aureus* and *Streptococcus* sp. isolates from patients with plaque-induced gingivitis. These compounds, present in chewing sticks and mouthwash, possess antibacterial, anti-inflammatory, and antioxidant properties. They also inhibit plaque formation and prevent periodontal disease by blocking the function of the glucosyltransferase enzyme.	[73]
*Streblus asper*	From the aerial bark, compounds such as n-triacontane, β-sitosterol, stigmasterol, tetraiacontan-3-one, oleanolic acid, and botulin are found. Additionally, flavonoids and lignans are derived from the heartwood.	Significantly reduced *S. mutans* colonies; effectiveness against *P. gingivalis* and *A. actinomycetemcomitans* colonies. These results revealed that extract is able to inhibit in vitro subgingival biofilm formation and reduce the numbers of *P. gingivalis, A. actinomycetemcomitans* and total bacteria.	[74]
*Terminalia chebula*	Fruit. Poluphenols, terpenes, anthocyanins, flavonoids, alkaloids, glycosides	Mouth rinses, toothpaste. Antibacterial properties against *A. actinomycetemcomitans* and *S. mutans*, anti-inflammatory and antioxidant properties.Prevents and treats dental caries and gingivitis.	[92]
*Thymus vulgaris*	Thyme essential oil major compounds were found as thymol, α-thymol, camphene, caryophyllene, humulene, α-terpineol and ρ-cymene.	*T. vulgaris* essential oil holds promise for inclusion in toothpaste formulations, EO exhibits antimicrobial activity against clinical isolates of pathogenic bacteria, including *S. mutans*, *P. gingivalis*, *S. pyogenes*, and *C. albicans.*	[76]
*Vaccinium macrocarpon*	Anthocyanins, flavonols, proanthocyanidins	Inhibits the colonization of bacterias such as *P. gingivalis*, *F. nucleatum* Prevents *P. gingivalis* from adhering to various proteins, which could lead to periodontal disorders. Antibacterial properties against *S. mutans*, *S. sobrinus*, *S. oralis.*	[93,94]
*Vitis vinifera*	Phenolic compounds	Anti-inflammatory, antioxidant, cytoprotective properties. Antibacterial, antifungal and antiviral activity against oral infections. Controls the bacterial-induced inflammatory response and oxidative stress imbalance in periodontal diseases.	[78]

**Table 5:** Plants with anti-dental caries properties: phytochemicals and clinical trial studies.

Plant Specie	Compound(s)	Study Model	Administration	Effect	References
*Allium sativum *	Allicin, Alliin, Diallyl trisulfide, S-allyl cysteine, Allyl mercaptan, Ajoene.	Human clinical trial; 200 subjects (20–60 years old). Human clinical trial, 90 children (4–6 years old).	Oral consumption; 8 tablets (300 mg AGE powder/tablet) during 18 months.	Consumption of AGE tablets to be effective as a preventive measure of periodontitis. Antibacterial activity. Garlic extract can be used safely for irrigation of root canals of primary molars.	[101,102]
*Aloe vera*	Anthraquinones	Randomized clinical study; 72 extracted human molars. Comparative clinical trals: *A. vera*, *O. sanctum*, chlorhexidin mouthwashes.	Toothpaste containing 1000–1450 ppm fluoride and *A. vera.* Mouthwash with *A. vera*; daily use, for 30 days.	Improvement in the enamel density values after demineralization. Usage of *A. vera-* mouthwash showed a significant reduction in plaque, gingival and bleeding indices over 30 days.	[103,104]
*Rosmarinus officinalis*	Borneol, camphor, limonene, camphene, pinene, cineol, myrcene, verbenone and caryophyllene.	Clinical study/n = 110 subjects	Toothpaste of daily use.	Reduced risk of gingival bleeding, prevented the increase in plaque formation.	[105,106]
*Ocimum sanctum*	Caryophyllene, pinene, copaene Civsilineol, Civsimavatine, Isothymonin, Apigenin, Rosavinic acid, Eugenol and Linoleic acid.	Triple-blinded trial: 84 participants (14–15-year-old). Comparative clinical trals: *O. sanctum*, and *A. vera* CHX mouthwashes.	Toothpaste, daily use for 21 days. Mouthwash added with *O. santum*; daily use, 30 days.	Significant reduction in the plaque and gingival scores. Antigingivitis effect. Usage of *O. sanctum* mouthwash reduced plaque, gingival and bledding indices over 30 days.	[104,107,108]
*Salvadora persica*	Caryophellene, cineole (eucalyptol), caryophellene, pinene	Comparative study (clinical trial; 40 students (16–18 years old). Miswak toothpaste vs. Miswak mouthwash or ordinary toothpaste. Comparative study (clinical trial; 60 girls (18–22 years old).	Twice daily (morning and before sleeping) for 2 weeks. *S. persica* extracts /10 g/100 mL) Daily use, during 6 months.	Mouthwash group presents antigingivitis, anticariogenic, antiplaque, whitening properties; orthodontic chain preservation and promotion of gingival wound healing.	[44,71,109]
*Camellia sinensis*	Polyphenols, epigallocatechin gallate (EGCG).	In vivo study; 90 children (4–6 year-old).	Extracts in the form of a gel for 2 weeks and analysis after breakfast, without tootbrush/toothpaste use.	Extracts diminished salivary *S. mutans* levels and has antibacterial activity against predominant cariogenic bacteria.	[25,110,111,112]
*Vitis vinifera*	Pro-anthocyanidins catechin, epicatechin and procyanidins.	Comparative study of pomegranate and guava extracts; 40 children (8–10 years old). Comparative study: 80 children of 8–15 years of age.	Aqueous extracts GSE (100 mg/mL) added to mouthwash was used (7 days)	Prevention of dental caries. Reduction in the oral streptococci counts. Inhibition of *S. mutans* biofilms. Plaque reduction (antioxidant and phytochemical properties).	[15,16,17,18]
*Punica granatum*	Plyphenolic flavonoids (punicalagins and ellagic acid), tannins.	Comparative study of grape seed and guava extracts; 40 children (8–10 years old). Comparative study: 80 children of 8–15 years of age.	Aqueous extracts (100 mg/mL) added to mouthwash was used (7 days). Fruit extracts from *P. granatum* and *T. chebula* added to mouthwash.	Reduction in the oral streptococci counts. Plaque reduction (antioxidant and phytochemical properties).	[16,18]
*Psidium guajava*	Saponins, tannins, flavanoids and alkaloids. Guaijaverin.	Comparative study of pomegranate and grape seed extracts; 40 children (8–10 years old).	Aqueous extracts (100 mg/mL) added to mouthwash was used (7 days).	Activity vs. *S.* *aureus*, *E.coli*, *C. albicans* and *S. mutans*	[18]
*Terminalia chebula*	Chebulic acid, Chebulagic acid, Corilagin, and Gallic acid.	Comparative study of pomegranate and grape seed extracts; 80 children (8–15 years old).	Mouthwash used daily, for 15, 30 days.	Diminished count of *S. mutans* in saliva (microbiologic assay).	[16]
*Lentinula edodes*	Eritadenina, Lentinan, Emitanina, Quitina, Ergosterols	In vivo studies/ 65 healthy adults.	Rinsing with extract (<5.000 Da) of shiitake (*L. edodes*) for 14–15 weeks.	Metabolic activity of dental plaque was reduced. But no reduction in plaque scores and no inhibition of the production of organic acids in plaque.	[113]
*Cichorium intybus*	Phenylpropenoids, anthocyanins, flavonoids, polysaccharides (such as inulin), sesquiterpenoids, triterpenoids, proteins, steroids, lipids, caffeine, and organic acids.	Double-blind, controlled clinical trial (40 patients with periodontitis).	1 g (capsules), twice daily, over 8 weeks.	It had potent antimicrobial activity against *S. mutans;* antioxidant and antiproliferative activity. Chicory leaf extract may be helpful in controlling periodontal status.	[114,115]
*Azadirachta indica*	Hexadecanoic acid, Hentriacontane, Phytol.	Comparative clinical study (60 patients) Neem-based toothpaste vs. probiotic-based toothpaste.	Neem-based toothpaste, used for 60 days.	The neem-based toothpaste showed antimicrobial activity in terms of reduction in the level of *S. mutans*.	[116]
*Streblus asper*	Amyrin acetate, sitosterol, streblosid, lupeol acetate, diol, Sioraside, amyrin, mansonin, Threo-streblusol B, streblusquinone, streblusol A, E, C y D, triacontane, tetraiacontan-3-one, oleanolic acid, botulin.	Randomized controlled trial: 76 subjects (14–18 years old).	Mouthwash used for 60 sec. then saliva sample collection (0, 2, 30, 60 and 120 min).	Bactericidal activity towards *S. mutans.*	[117]

min: minutes; sec: seconds; ml: milliliter; mg: milligrams; mg/mL: milligrams per milliliter; ppm: parts per million.

**Table 6:** Minimum inhibitory concentration (MIC) values in mg/mL caused by diverse plants against cariogenic bacteria.

Plant Extract	Pathogen	MIC Value (mg/mL)	Reference
*Melaleuca alternifolia*	*Streptococcus* spp.	1.2–20	[63]
*Allium sativum*	*Streptococcus mutans*	0.5–32.0	[63]
*Psidium guajava*	*S. mutans*	0.076	[63]
*Glycorrhiza glabra*	*S mutans*	<12.5	[63]
*Bauhinia purpura*	*S. mutans*	0.029	[63]
*Viburum foetens*	*S. mutans*	0.08	[63]
*Madhuca longifolia*	*S. mutans*	0.0183–0.0212	[63]
*Morus alba*	*S. mutans*	0.008	[63]
*Rosmarinus officinalis*	*S. mutans*	4.0	[63]
*Terminalia chebula*	*S. mutans*	0.076	[63]
*Vitis coignetiae*	*S. mutans*	7.50	[63]
*Camellia sinensis*	*S. mutans*	80.0	[112]
*Camellia sinensis*	*Streptococcus sanguinis*	80.0–250.0	[112]
*Bauhinia purpura*	*Lactobacillus acidophilus*	0.0202	[63]
*Laurus nobilis*	*Candida albicans*	0.25	[63]
*Psidium guajava*	*C. albicans*	0.152	[63]
*Vitis vinifera*	*Porphyromonas gingivalis*	0.48	[123]
*Pongamia pinnata*	*S. mutans*	0.20	[122]
*Acacia catechu*	*S. mutans*	0.20	[122]
*Aloe vera*	*S. mutans*	0.25–3.0	[121]
*Equisetum arvense*	*S. mutans*	0.25–3.0	[121]
*Mimosa tenuiflora*	*S. mutans*	0.25–3.0	[121]
*Lippia graveolens*	*S. mutans*	0.25–3.0	[121]
*Syzygium aromaticum*	*S. mutans*	0.25–3.0	[121]
*Aloe vera*	*Streptococcus sobrinus*	2.0–3.0	[121]
*Equisetum arvense*	*S. sobrinus*	2.0–3.0	[121]
*Melaleuca tenuiflora*	*S. sobrinus*	2.0–3.0	[121]
*Lippia graveolens*	*S. sobrinus*	2.0–3.0	[121]
*Syzygium aromaticum*	*S. sobrinus*	2.0–3.0	[121]

**Table 7:** MICs and MBCs in mg/mL of antimicrobials commonly used in dentistry.

Antimicrobial Agent	MIC (mg/mL)	MBC (mg/mL)	Reference
Sodium fluoride	0.625	2.5	[127]
Tea polyphenol	1.25	2.5	
Chlorhexidine	0.0025	0.0025	
Penicillin	0.000047	0.000094	
Metronidazole	0.250	0.5	

MIC: minimum inhibitory concentration, MBC: minimal bactericidal concentration.

**Table 8:** Diverse natural products nanoencapsulated into nanoparticles.

NPs Based	Plant Encapsulated	Microorganism Effect	MIC	Reference
AgNPs	*Punica granatum* associated with CaGP	*S. mutans* and *C. albicans*	ND	[130]
AgNPs	Collagen	*S. mutans*	125 ppm	[143]
CuNPs	*Moringa oleifera*	*E. coli*, *Klebsiella pneumonia*, *E. faecalis, S. aureus* and *C. albicans*	0.3 mg/mL	[144]
SiNPs	Green tea powder	*S. mutans*	600 μg/mL	[145]
AgNPs + chitosan + fluoride	*Camellia sinensis*	ND	ND	[146]
AgNPs	*Ulva flexuosa, Neochloris oleoabundans* and *Fucus vesiculosus*	*B. subtilis,* *S. aureus*, *E. coli*, and *Pseudomonas aeruginosa*	ND	[147]
SnCl_2_	*Citrullus lanatus*	*S. mutans*	0.531–1.176 μg/mL	[148]
Nanoliposomes	Aqueous plant extracts of licorice, ginger, pomegranate, and rose	*S. mutans*	2.0–2048 µg/mL	[149]

AgNPs: silver nanoparticles; AuNPs: gold nanoparticles; CuNPs: copper nanoparticles; CuONPs: copper oxide nanoparticles; CsNPs: chitosan nanoparticles; TiO2NPs: titanium dioxide nanoparticles; SiNPs: silica nanoparticles; SnCl_2_: stannous chloride nanoparticles; MIC: minimum inhibitory concentration; μg/mL: micrograms per milliliter; ppm: parts per million; ND: not described.

**Table 9:** Oral coatings’ effectiveness against cariogenic microorganisms.

Coating	Treatment	Microorganism	Reference
Tooth-Coating ZIF-C	GIC contains zinc mixture. Randomized controlled trial.	*S. mutans*	[158]
Coatings Ni-Co-ZrO_2_	Electrodeposition on the micro-surface of spindle hook teeth	-	[159]
Coating of fluoridated apatite (FAp)	LAB.	*S. mutans*	[160]
Coating of magnesium (Mg)	Alloys with ZrNPs.	*Actinomyces* spp., *P. gingivalis* and *C. albicans*	[161]
Coating of synthesis of zirconia (ZrO_2_)	Tangerine-mediated synthesis of ZrO_2_ NPs as potential protective dental coatings.	*Streptococcus*, *E. coli* and *Bacillus subtilis*	[162]
Coatings of salvia	A randomized controlled trial.	*S. mutans*, *P. intermedia* and *P. gingivalis.*	[163]
Coatings of Hidroxiapatit-TiO_2_	Dental implants.	*S. mutans*, *S. aureus* and *C. albicans*	[164]
Polyhydroxyalkanoates coatings	Dental implants.	*S. aureus* and *E. coli*	[165]

NPs: nanoparticles; GIC: glass ionomer cement; ZIF-C: zinc caredyne coatings; ZrO_2_: zirconia; TiO_2_: titanium dioxide; LAB: laser-assisted biomimetic process; MIC: minimum inhibitory concentration.

## Data Availability

Not applicable.
